# Creating Well-Being: Increased Creativity and proNGF Decrease following Quadrato Motor Training

**DOI:** 10.1155/2015/275062

**Published:** 2015-06-07

**Authors:** Sabrina Venditti, Loredana Verdone, Caterina Pesce, Nicoletta Tocci, Micaela Caserta, Tal Dotan Ben-Soussan

**Affiliations:** ^1^Department of Biology and Biotechnology “Charles Darwin”, Sapienza University of Rome, 00185 Rome, Italy; ^2^Institute of Biology and Molecular Pathology, National Research Council (CNR), 00185 Rome, Italy; ^3^Department of Movement, Human and Health Sciences, Italian University for Sport and Movement, Rome, Italy; ^4^Research Institute for Neuroscience, Education and Didactics, Patrizio Paoletti Foundation, Via Cristoforo Cecci 2, Santa Maria degli Angeli, 06081 Assisi, Italy

## Abstract

Mind-body practices (MBP) are known to induce electrophysiological and morphological changes, whereas reports related to changes of neurotrophins are surprisingly scarce. Consequently, in the current paper, we focused on the Quadrato motor training (QMT), a newly developed whole-body movement-based MBP, which has been reported to enhance creativity. Here we report the effects of 4 weeks of daily QMT on creativity and proNGF level in two interrelated studies. In Study A, we examined the effects of QMT compared with a walking training (WT) in healthy adults, utilizing the alternate uses task. In contrast with the WT, QMT resulted in increased creativity. In addition, the change in creativity negatively correlated with the change in proNGF levels. In Study B, we examined QMT effects on creativity and additional metacognitive functions in children, using a nonintervention group as control. Similar to Study A, following QMT, we found a negative correlation of proNGF with creativity, as well as working memory updating and planning ability. Together, the current results point to the relationship between increased creativity and decreased proNGF following MBP. Thus, the current research emphasizes the importance of widening the scope of examination of “*MBP in motion*” in relation to metacognition and well-being.

## 1. Introduction

Creativity is considered important both for personal well-being and social growth [1]. It is an important function through which we can cope with significant challenges in our environments in novel and appropriate ways [2]. The lexeme in the English word creativity comes from the Latin term creō, meaning “to create, make.” Thus, creativity means bringing into being, as it involves generation of novelty and transformation of existent information [1]. In its wider meaning, creativity can be understood as the ability to adapt cognitive processing strategies to face new and unexpected conditions and is thus closely related to efficient problem-solving and coping with stress, resulting in increased well-being [3–6]. Research examining the effects of mindful training on creative performance has been relatively scarce and mainly focused on sitting meditations. While some studies found evidence for a strong positive impact of meditation practice on creativity [7], others found only a weak association or no effect at all [8, 9]. Research examining the effect of whole-body movement-based mind-body practices (MBP) on creativity is lacking, and the current report is intended to start filling this gap. To this aim we examined the Quadrato motor training (QMT), a whole-body movement practice, which has been recently found to increase creativity and emotional well-being [10, 11]. In addition, a single session of QMT was found to improve spatial cognition and reflectivity [12] in contrast to two different control groups controlling separately for cognitive and motor load.

In addition to the QMT-induced effects on creativity and well-being, QMT has been found to increase alpha (8–13 Hz) synchronization [10, 12]. Since decreased alpha synchronization [13], in parallel to increased proNGF levels, was found in different health-related disorders, such as Alzheimer's disease [14], in the current paper we expanded our examination from electrophysiological to physiological effects of QMT by analyzing the effects of 4 weeks of daily QMT on creativity and proNGF level in healthy adults and children.

Neurotrophins (NTs) are central players of many aspects of the developing and adult nervous system, including neuronal differentiation, synaptogenesis, and synaptic plasticity. Nerve growth factor (NGF) was the first neurotrophin to be described [15]. It is involved in the regulation of naturally occurring neuronal death, synaptic connectivity, fiber guidance, and dendritic morphology of neuronal populations in the peripheral (sympathetic) and central nervous system (for a review see [16]). Early observations by Aloe and coworkers have revealed that NGF is actively synthesized by the submandibular salivary gland in mice, overproduced following aggressive behaviour and released in the bloodstream under stressful conditions [17]. Its relevance in the limbic areas involved in mood, cognition, and circadian activities as well as in the neuroendocrine system suggested that it might regulate the individual responses to stress by modulating the activity in networks that determine how plastic changes influence mood and behaviour [18, 19].

NGF is produced by neurons as a precursor,* proNGF*, and released in the synaptic space, where it undergoes cleavage and maturation. Finally, the mature form is internalized by the postsynaptic membrane through binding to the high affinity TrKA (tropomyosin-related kinases) receptor (for a review see [20]). In addition, proNGF is believed to contribute to neuronal plasticity and neural connection shaping of the adult brain, a function that is likely mediated by the complementary ability of proNGF to promote apoptosis during development (for a review see [21]). Consistent with this, proNGF gained large attention when it became clear that not only it is the most prevalent form in the human brain, but also it significantly increases in the brains of Alzheimer's patients and of individuals with several degrees of cognitive impairment [14, 22]. Thus, from the psychoneuroimmunological point of view, motor-mental practices could be important means to contribute to the proper activation of neurotrophic pathways aimed at facilitating coping with stressful conditions and at increasing creativity and well-being.

Although a large literature is available on the correlation between training, environmental enrichment, and increased brain-derived neurotrophic factor (BDNF) levels and their neuroprotective effects in both humans (see [23] and the references therein and [24]) and animal models [25–27], very few reports have explored the relationship with proNGF. Likewise no studies that investigate the connection between proNGF level and creativity are available to date, and no research that we are aware of examined whether whole-body cognitive training practices influence the relationship between the two. Consequently, the aim of the present study was to examine the effects of QMT on three main parameters: creativity, salivary proNGF, and the correlation between them. To this end we conducted two interrelated studies, examining the effects of 4 weeks of daily QMT on creativity and proNGF in healthy adults and children. In addition, we examined the relationship between change in proNGF level and creativity. In the developmental study we extended behavioral testing to further high-level and metacognitive functions to obtain a more comprehensive view on the type of cognition affected by the QMT.

## 2. Materials and Methods

### 2.1. Study A

#### 2.1.1. Participants and Design

A total of 40 female university students (mean age ± SD: 30 ± 5.5 years) were enrolled in the study, none of whom had practiced QMT before. All were healthy with no medical history that might affect their performance. The study was conducted at the Sapienza University and was approved by the CNR Research Ethics and Bioethics Advisory Committee. Upon arrival, the participants signed a written informed consent. Subsequently, saliva samples were collected and a cognitive task tapping creativity was performed. All data were collected both before and after 4 weeks of daily practice. Participants were randomly allocated to one of the two groups: (1) Quadrato motor training (QMT, 3 choices and whole-body response) and (2) walking training (WT, 1 choice and whole-body response). Although originally the initial group sizes were identical (*n* = 20), the final number of participants finishing the 4-week training varied between the groups (QMT, *n* = 13; WT, *n* = 6). The QMT has been described in detail elsewhere [10]. Here, we describe it briefly.

#### 2.1.2. Training Groups

*Quadrato Motor Training (QMT)*. The QMT requires standing at one corner of a 0.5 m × 0.5 m square and making movements in response to verbal instructions given by an audio tape recording. There are 3 optional directions of movement. The instructions direct participants to keep the eyes focused straight ahead and hands loose at the side of the body. They are also told to immediately continue with the next instruction and not to stop in the case of mistakes. At each corner, there are three possible directions to move in. The training thus consists of 12 possible movements. The daily training consisted of a sequence of 69 commands lasting 7 minutes. Two important variables that were addressed in motor learning studies are limb velocity and the decision regarding the responding limb [28, 29]. In order to control these parameters, we used a movement sequence paced at a rate of an average of 0.5 Hz (similar to a slow walking rate) and instructed the participants to begin all movements with the leg closest to the center of the square.

*Walking Training (SMT)*. The participants belonging to the WT group were instructed to make successive steps (one step following the auditory stimulus) using the same QMT pace, duration, and auditory cue, but their movement was free in space (not within a square). This group also practiced with the same recordings of auditory stimuli as the QMT group. However, while the QMT group was told that each number represented a different corner of the square, the WT group was told to simply walk beginning at a certain location and to continue in response to the instructions. That is, regardless of the number specified on the tape, they always made a step. This reduced the uncertainty regarding the direction of the movement, compared to the QMT group. The WT group thus provided a control performing a task with similar motor demands, but with reduced cognitive demands.

#### 2.1.3. Cognitive Examination: The Alternate Uses (AU) Task

The AU task is an established psychometric creativity test which provides measures for both fluency and flexibility [30, 31]. This task has been previously used to study changes in ideational flexibility following whole-body training [32, 33]. In this task, the participant is required to name as many different ways as possible in which a given item might be used within a 1-minute time frame. For example, a shoe can be used to walk with or can serve as a drum. Two basic measures were computed from the AU task:* ideational fluency*, defined as the total number of ideas generated, and* ideational flexibility*, defined as the tendency to generate a heterogeneous pool of responses or to use a variety of categories and themes when producing ideas [34]. Flexibility conveys information that is not conveyed by fluency [34, 35]. Here, the* ideational fluency* score was defined as the mean number of uses given by the participant for the three items. On the basis of all the uses made by the participants, 10 independent categories were defined across all the items. These included broad categories of usage such as “a weapon” or “a costume.” The* ideational flexibility* score was defined as the mean number of different categories employed by the participant across all three words presented [36]. Hence, in order to calculate the flexibility score, all responses for a given item were first divided into the different independent categories. For additional details see [10, 12].

#### 2.1.4. proNGF Examination

*Saliva Sample Preparation*. Unstimulated whole saliva samples were collected by passive drool and stored at −80°. Prior to electrophoresis, samples were subjected to vortex for 30 seconds and then centrifuged at maximum speed for 15 minutes. Saliva supernatants were transferred to fresh tubes, protease inhibitor cocktail (Roche) was added, and total protein concentration was determined. In order to measure the level of salivary NGF, we utilized western blotting and not ELISA assays, since this last technique does not allow discriminating between precursor and mature NGF [37]. Samples were examined in triplicate to take into consideration potential variability due to saliva flow rate, before the training and following 4 weeks of daily QMT practice.

*Western Blot Analysis*. 50 *μ*g of total proteins were resolved by SDS-PAGE on 4–15% precast gradient gels, transferred onto 0.2 *μ*m PVDF membranes by Trans-Blot Turbo Blotting System (BIO-RAD), and hybridized with anti NGF (Santa Cruz Biotechnology, sc-548, 1 : 1000), followed by anti-rabbit secondary antibody (Jackson ImmunoResearch, 111-035-003, 1 : 20000). The bands corresponding to proNGF were quantified with Image Lab software and normalized to the most intense band visible on the membrane in the protein loading control.

#### 2.1.5. Statistical Analysis

To answer the first question, that is, what are the effects of whole-body training on functional and molecular aspects of cognition, we submitted fluency and flexibility scores of creativity as well as NGF level to a Group (QMT versus WT) × Training (pre versus post) mixed-model analysis of variance (ANOVA). Since baseline differences were observed in flexibility score, we also analyzed the data with the regressor variable method, that is entering the post-treatment measure as the dependent and the baseline measure as the covariate in a univariate ANOVA model. To answer the question whether intervention-induced changes in proNGF level represent a correlate of changes in creativity, the strength of the correlation between pre-post Δ values of proNGF level and of indices of fluency and flexibility was estimated by means of bivariate correlation analysis (Pearson's* r*).

### 2.2. Study B

#### 2.2.1. Participants and Design

A total of twenty healthy children participated in this study (mean age ± SD: 10, 4 ± 0.4 yrs), none of whom practiced QMT before. A similar design applied as in Study A, except that in this study; the control group consisted of subjects that did not undergo any form of intervention. Among the contacted children, 12 children volunteered and were divided into two groups: one performed the QMT (*n* = 5) and the other one represented the no training control (*n* = 7). Both groups were retested after the end of the intervention period. The study took place in two schools. A written informed consent was signed by all parents before entering the study. The study was approved by the CNR Research Ethics and Bioethics Advisory Committee. Following a month, 12 healthy children finished the training (QMT, *n* = 5), control group (C, *n* = 7).

Cognitive tests were administered by a trained experimenter in the morning, within the same week in the same order and not preceded by physical education lessons to avoid acute exercise effects on cognitive function [38]. Cognitive tests were administered following the saliva sampling. Since the number of children was small, instead of counterbalancing, we have decided a fixed testing sequence for all children, with the saliva samples collection first.

#### 2.2.2. Cognitive Examination

Cognitive testing comprised tests of creative thinking and of executive function, lasting about 20–25 min and 30–35 min, respectively. Creative thinking: (1) Torrance Test of Creative Thinking (TTCT); executive function: (2) random number generation (RNG) task and (3) planning and attention subtasks of the Cognitive Assessment System (CAS).

*Torrance Test of Creative Thinking*. The Italian version of the Torrance Test of Creative Thinking (TTCT), Figural Form A [39], was group-administered at school. The TTCT Figural Form A is designed for individuals in kindergarten through graduate school and beyond. It consists of three timed pencil and paper picture construction and completion activities lasting 10 minutes each, with one-minute break between tasks for a total working time of about 30 minutes. According to testing guidelines, the administrator invited the examinees to enjoy these activities and created a playful problem-solving atmosphere to minimize threatening feelings linked to a performance-oriented climate.

*Activity I: Picture Construction Task.* Children had to construct a picture using a darkened curve shape (jellybean or teardrop) provided on the page as a stimulus which must be integrated in the picture construction.

*Activity II: Picture Completion.* Children had to use 10 incomplete figures to make a figure or object drawings to the incomplete figures, avoiding usual and obvious completions.

*Activity III: Parallel Lines.* Children had to use 30 pairs of straight lines drawn on three pages to make an original picture out of each pair of lines, overcoming the tendency to perceive the same stimuli in the same way.

*Data Coding and Scoring*. Scoring of the TTCT was performed by a trained investigator based on three subscales of norm-referenced measures: fluency, flexibility, and originality. Fluency was scored by the number of figural images produced by the examinees; it reflects the ability to generate a large amount of relevant ideas. Flexibility was scored by the variety of categories of relevant responses. Originality was scored by the number of statistically infrequent responses on the basis of normative data; it reflects the ability to produce uncommon or unique ideas. According to the TTCT manual, raw scores were converted into standard scores to have comparable ranges for fluidity, flexibility, and originality.

*Random Number Generation Task*. This is a test that taps executive cognitive function and is feasible also with children [40]. The participants were tested individually. They were told that the RNG is a game involving numbers and were instructed to verbally generate a random sequence of numbers between 1 and 10 to each beat of a 70-beat sequence with an interbeat interval of 1.5 seconds. Randomness was explained by means of an age-appropriate instruction including a “hide-and-seek” type game [40]. Prior to data collection by tape recording, participants performed a familiarization trial of 70 numbers and could ask questions concerning the test. Both the omission of a number generation in correspondence of one tone and the production of numbers lower than 1 (0) or higher than 10 (11, 12, etc.) were considered errors and discarded. If errors exceeded a predefined maximum threshold of five, the entire block was repeated. The randomness of the sequence of numbers was measured by means of 18 different indices described by Towse and Neil [41]. Among those, five indices were selected as they reflect two components of executive function: inhibition of mental routines (turning point index (TPI), adjacency score (Adj), and runs score (Runs)) and working memory updating (redundancy score (Red), and mean repetition gap (MeanRG)).

The TPI is a ratio between the real frequency of turning points between ascending and descending series of numbers (i.e., the response change between the digits “2” and “5” in a hypothetical sequence “9, 7, 2, 5, 6, and 8”) generated by the participant and their theoretical frequency in random responses. Turning points in random responses are assumed to be 2/3*∗*(*n* − 2), where *n* is the number of digits to be generated. A TPI lower than the optimal value of 100 indicates that participants produced more or fewer turning points than theoretically expected. The Adj measures the relative frequency of pairs of adjacent ascending or descending numbers (i.e., 7-8 or 4-3) as compared to the total number of response pairs produced by the participant. It ranges between 0% and 100% and reflects the habitual tendency to count forward or backward. The Runs score is an index of variability of the number of digits in successive ascending or descending runs. Counting from 1 to 9 and from 9 to 1 along the whole sequence of generated numbers leads to the highest Run value, whereas alternating ascending and descending pairs of digits as “4, 7, 9, and 2” will lead to lowest scores or even to null if this alternation is produced throughout the sequence.

The Red index reflects the unbalance of response alternative frequencies in a sequence that derives from a more frequent usage of given numbers than expected based on the theoretical frequency of each digit in random responses. In the present experiment, a perfect equality of response alternative frequencies corresponds to the generation of each number from 1 to 10 seven times each and would lead to a Red score of 0% (no redundancy), whereas repeating the same digit along the whole sequence would lead to a Red score of 100% (complete redundancy). The MeanRG is the mean number of responses given until each digit reoccurs calculated for all digits throughout the whole sequence (i.e., in the sequence “2, 8, 4, 6, 2, 9, 7, and 8”; the digits “2” and “8” reoccur with a mean gap equal to 4). If the participant regularly varies all possible digits throughout the sequence, then the MeanRG is high, whereas repeating one or more items much more frequently than theoretically expected leads to a low MeanRG value.

TPI, Adj, and Runs were merged into an average index of inhibition and Red and MeanRG into an average index of memory updating. High levels of TPI, but low levels of A comb and Runs correspond to a high inhibition ability, as well as high levels of Mean RG, but low levels of Red correspond to a high ability to update working memory. Thus before averaging, all indices were standardized and Adj, Runs, and Red were reversed.

*Cognitive Assessment System*. To assess children's cognitive performance, the Cognitive Assessment System (CAS) [42] (Italian version 2005) was used. The CAS consists of 12 subtests that assess 4 aspects of cognition: Planning, Attention, Simultaneous, and Successive processes (PASS theory) [43]. For the present study, because of school time constraints, participants only performed the Planning and Attention tasks which are the test performances most strongly relying on executive functions. Also, we did not collect test-retest reliability data, since acceptable to good reliability data are available for children of the age 5–10 years considered in this study [42].

*Planning Subtasks*. Planning is a cognitive process by which the individual determines, selects, and uses a strategy to efficiently solve a problem. The Planning scale is composed of three subtests. The first subtest, Matching Numbers, contains 4 items, each with 8 rows of numbers and 6 numbers per row, with numbers increasing in digit length every four rows. The child must locate and underline the two numbers in each row that are the same. The second subtest, Planned Codes, contains two items, each within a matrix of 7 rows and 8 columns of letters with empty boxes. A caption is presented that shows correspondence between letters and codes presented as a legend at the top of the page (i.e., A to OX and B to XX). The child's task is to fill in the empty boxes under each letter with the corresponding codes discovering their internal organization to solve the task. The last subtest, Planned Connections, contains 8 items. The first six require the child to join a series of numbers that are randomly distributed in space in a sequential order, with an increasing length of numbers to be connected. The last two items require the child to alternately connect numbers and letters serially (i.e., 1-A-2-B, etc.). The items are designed so that a child cannot complete a sequence by crossing one line over the other. To evaluate performance on the first two subtests, the raw score is the ratio of the accuracy and time to completion. For the third subtest, the raw score is the sum of the times required to complete each item. All these scores are then converted to an age-based standard score and summed to obtain a total scale value.

*Attention Subtasks*. The Attention scale is composed of three subtests that require the child to use focal attention to detect target stimuli and avoid distractions. The first subtest, Expressive Attention, is a Stroop-like test composed of three items that measures attention selectivity and interference control under time pressure. The first and the second items are without interference condition, while the third is with interference. There are two age-specific sets of items. For example, in the version for children 8 years and older, the noninterference conditions are reading color words (Blue, Yellow, Green, and Red), all written in dark ink and naming the colors of a series of rectangles (printed in blue, yellow, green, and red). In the interference condition, the words Blue, Yellow, Green, and Red are printed in a different color ink than the colors of the words' name and the child is instructed to name the color ink the word is printed in, rather than to read the word. Only this last item is used as the measure of attention. The second subtest, Number Detection, measures selectiveness and capacity to resist distraction under time pressure. It is comprised of pages of numbers where the child must underline the correct numbers among a large quantity of distracters in different formats. For example, the child must find a particular stimulus (the numbers 1, 2, and 3 printed in an open font) on a page containing many distracters (the same numbers printed in a different font style). The raw score is the ratio of the accuracy and the time to completion summed across the items. The third subtest, Receptive Attention, is a two-page subtest that measures the ability to focus and then shift attention between different stimulus dimensions under time pressure. On the first page, children of 8 years and older must identify and underline pairs of target letters that are physically identical (e.g., TT but not Tt), whereas on the second, pairs of letters that have the same name (e.g., Aa not Ba) are targets to be underlined. For all subtests, the raw score is the ratio of the accuracy and time to completion summed across items/pages. The raw score for each subtest is converted to an age-based standard score and summed to obtain a total scale value.

#### 2.2.3. Molecular Examination

For salivary sample preparation and western blotting analysis see Study A.

#### 2.2.4. Statistical Analysis

To answer the question regarding the effects of the QMT on cognitive performance and on neurobiological markers of brain health, a general linear model of multivariate analysis of variance (MANOVA) and subsequent ANOVAs were applied to the pre-post Δ values of fluency, flexibility, and originality dimensions of creative thinking, average indices of inhibition and working memory updating, and total scores of planning and attention, as well as on proNGF level. In the present nonequivalent control group design, baseline cognitive differences could not be excluded between groups, since participants were not individually randomized to the intervention or control condition. Thus, in addition to the change score method (Δ values), data were analysed with the regressor variable method, that is entering the post-treatment measure as the dependent and the baseline measure as the covariate. These methods were considered more appropriate to tap differential intervention effects than using a mixed model with group as between-participants factor and measurement time (pre- versus post-intervention) as repeated measures factor.

To answer the question of whether and to what extent intervention-induced changes in proNGF level represent a correlate of changes in cognitive efficiency, the strength of the correlation between pre-post Δ values of proNGF level and all above listed cognitive performance indices was estimated by means of bivariate correlation analysis (Pearson's* r*). In order to avoid that preexisting baseline differences among children could prevent from detecting correlations between intervention-related cognitive improvements and their potential neurobiological markers, bivariate correlations were also computed between post-intervention measures of proNGF level and all indices of cognitive efficiency. In order to control for multiple comparisons, we chose *P* < 0.007.

## 3. Results 

### 3.1. Study A

#### 3.1.1. Cognitive Results

**Figure 1:**
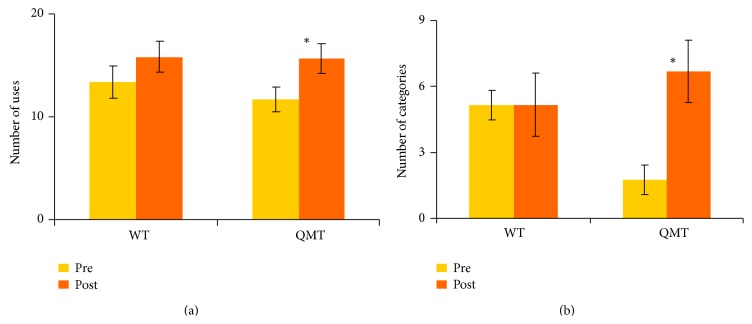
Change in creativity as a function of group and training for (a) ideational fluency and (b) ideational flexibility. Data are expressed as mean ± SEM; ^*∗*^ *P* < 0.05.

The first 2-way ANOVA designed to answer the question concerning the whole-body training-induced effects on fluency revealed a main effect for training (*F*(1,17) = 27.95, MSE = .35, and *P* < 0.01), with post-training being generally higher compared to pre-training (Figure 1(a)). Although the Group × Training interaction was not significant (*F*(2,15) < 1), fluency significantly increased only in the QMT group (*t*(12) = −7.21, *P* < 0.001) (Figure 1(a)). The second ANOVA which was conducted for flexibility yielded a significant Group × Training interaction (*F*(1,17) = 6.69, MSE = .82, and *P* < 0.05). Post-hoc analysis (*t*-tests) showed that for the QMT group, flexibility significantly increased (*t*(12) = −5.14, *P* < 0.001) in contrast to WT group which showed no change following training (Figure 1(b)).

The results of the univariate ANOVA performed on post-intervention fluency values with pre-intervention fluency values as covariates yield a significant effect only for the pre-intervention score entered as covariate (*F*(1,17) = 56.71, *P* < 0.001), with post-training being generally higher compared to pre-training in both intervention groups (Figure 1(a)). Instead, the results of the ANOVA performed on post-intervention flexibility values with pre-intervention flexibility values as covariates yield a significant effect for group (*F*(1,16) = 6.72, MSE = 1.6, and *P* < 0.05) and not only for the pre-intervention score entered as covariate (*F*(1,16) = 9.75, *P* < 0.01).

#### 3.1.2. Molecular Results

**Figure 2:**
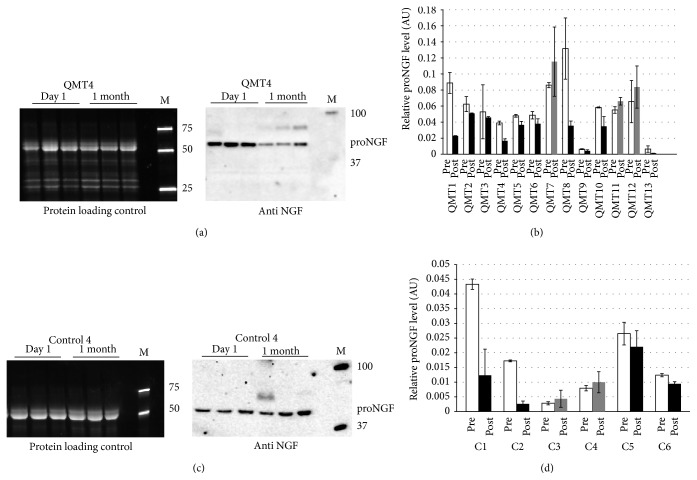
Western blot analysis of proNGF level in healthy adults. (a) Representative gel and blot for a typical QMT participant (QMT4). Left panel: picture of the gel after electrophoresis, showing total salivary proteins. Right panel: immunoblot showing proNGF. M: molecular size marker (values in kD). (b) Histograms represent relative proNGF level for all the QMT participants: each histogram indicates the average of the 3 proNGF values normalized to the most intense band present in the corresponding lane of the protein loading control. Data are expressed as mean ± SD. White (pre), relative proNGF level before starting the training; black (post), relative proNGF level after 1 month of training: the value is lower than before the training; grey (post), relative proNGF level after 1 month of training: the value is higher than before the training. (c) As in (a), but for a typical control participant (Control 4). (d) As in (b), but for all the control participants.

The third ANOVA which was performed on NGF values revealed no main effect for Training or Group × Training interaction. The results of the western blot analysis are shown in Figure 2. Figures 2(a) and 2(c) show representative gels for a typical QMT and control participant, respectively; Figures 2(b) and 2(d) show the quantification for all the QMT and control participants, respectively. It can be easily observed that following daily QMT practice for a duration of a month there was a trend of proNGF level decrease for the majority of participants (77% of the participants). This trend of proNGF decrease was further observed in 67% of the WT group.

#### 3.1.3. Correlation between Cognitive and Molecular Results

**Figure 3:**
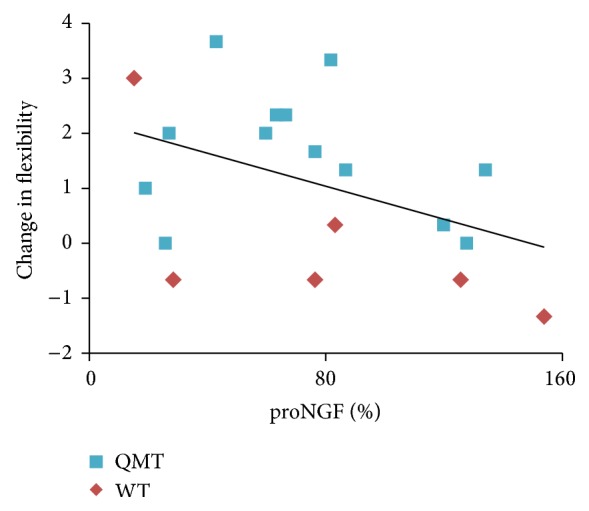
Significant correlation between change in ideational flexibility and proNGF level (*n* = 19). Change in ideational flexibility, calculated by the subtraction of pre- from post-training, was negatively correlated with the change in NGF level, calculated as percentage (*r* = −0.43, *P* < 0.05, and *n* = 19).

We then investigated whether the increased ideational flexibility was correlated with the observed changes in proNGF level by means of a Pearson's correlation. Change in ideational flexibility was calculated by subtracting the number of independent categories before training from the number of categories after training. In order to calculate change in proNGF we calculated the percentage by subtracting post- from pre-training proNGF. As can be seen in Figure 3, change in ideational flexibility was significantly and negatively correlated with change in proNGF (*r* = −0.43, *P* < 0.05, *n* = 19).

Taken together the results of Study A show that similar to the effects of one QMT session [10], 4 weeks of daily QMT practice induce increased ideational flexibility in adults. On the other hand, contrary to one session of QMT, 4 weeks of daily QMT practice may further enhance ideational fluency. However, a differential effect of QMT as compared to simple WT emerged only for flexibility. In addition, training-induced changes in ideational flexibility are negatively correlated with decrease of proNGF. A limitation of this study is that we used only one measure of cognitive function, whereas in exercise and cognition research it is recommended to use multiple measures of the target high-level cognition to assess the construct more broadly and understand the task specificity of the effects [44]. Furthermore, limiting the assessment of QMT effects to the adult population does not allow appreciating the educational potential at developmental age, where exercise is expected to have wider ranging effects of larger size [45]. To overcome these limitations and in order to better dissect the link between molecular and cognitive changes, we introduced the design of Study B, in which we examined the effects of QMT (compared to a control group performing no training) in children, and used a set of cognitive tasks, validated for children, that target not only creativity, but also another relevant metacognitive function (i.e., planning) and further components of higher-level cognition (i.e., working memory updating and executive attention) that contribute to, but do not overlap with, creative thinking and planning.

### 3.2. Study B

#### 3.2.1. Cognitive Results

The effect for group of the MANOVA performed on pre-post Δ values of cognitive performance and concentration of neurobiological markers as a function of group approached significance (*P* = .063). Subsequent ANOVAs did not show any significant difference (.313 ≥ *P* ≥ .960) for all dependent variables except for Attention (*F*(1,10) = 10.99, *P* = .008, and *η*_*p*_^2^ = .52), where the pre-post Δ values were significantly higher in the experimental than in the control group (8.0 versus 1.6). However, the results of the ANOVAs performed on post-intervention values with pre-intervention values as covariates did not yield any significant effect for group (.349 ≥ *P* ≥ .926) for all dependent variables. The ANOVA performed on post-intervention scores of Attention also was nonsignificant (*P* = .711), while the effect of pre-intervention score entered as covariate was significant (*F*(1, 9) = 8.42, *P* = .018, and *η*_*p*_^2^ = .48), thus indicating that baseline differences between groups were responsible of the significant group effect found in the analysis.

#### 3.2.2. Molecular Results

**Figure 4:**
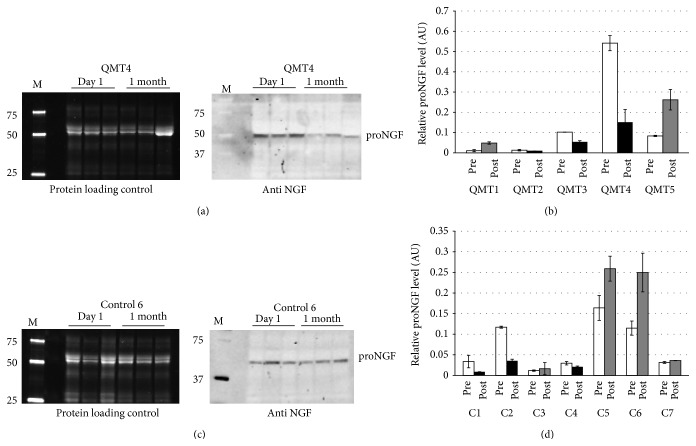
Western blot analysis of proNGF level in healthy children. (a) Representative gel and blot for a typical QMT participant (QMT 4). Left panel: picture of the gel after electrophoresis, showing total salivary proteins. Right panel: immunoblot showing proNGF. M: molecular size marker (values in kD). (b) Histograms represent relative proNGF level for all the QMT participants: each histogram indicates the average of the 3 proNGF values normalized to the most intense band present in the corresponding lane of the protein loading control. Data are expressed as mean ± SD. White (pre), relative proNGF level before starting the training; black (post), relative proNGF level after 1 month of training: the value is lower than before the training; grey (post), relative proNGF level after 1 month of training: the value is higher than before the training. (c) As in (a), but for a typical control participant (control 6). (d) As in (b), but for all the control participants.

The results are shown in Figure 4. Figures 4(a) and 4(c) show representative gels for a typical QMT and control participant, respectively; Figures 4(b) and 4(d) show the quantification for all the QMT and control participants, respectively.

#### 3.2.3. Correlation between Cognitive and Molecular Results

**Figure 5:**
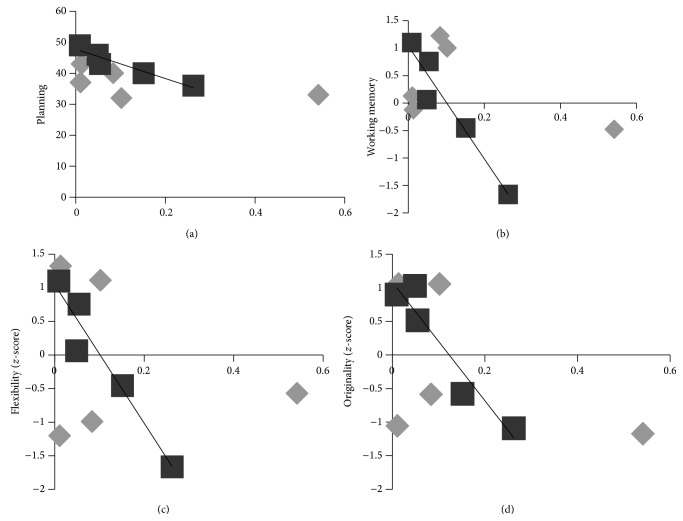
Significant correlations between proNGF level and cognitive performance following 4 weeks of daily QMT (*n* = 5) for (a) planning (*r* = −.95, *P* = .005); (b) working memory updating average index (*r* = −.96, *P* = .004); and (c) flexibility and (d) originality dimensions of creative thinking, for pre (gray) and post (black) QMT. Regression line is shown for post-QMT.

Results of the bivariate correlation analyses run on pre-post Δ values did not yield any significant association between change scores of proNGF concentration and change scores of cognitive performance neither for the intervention group (.93 ≥ *P* ≥ .14) nor for the control group (.89 ≥ *P* ≥ .23). Similarly, all correlations computed on pre-intervention values were nonsignificant for both the experimental (.73 ≥ *P* ≥ .25) and the control groups (.99 ≥ *P* ≥ .07). Instead, the analysis performed on pos-tintervention values showed very high and significant negative correlations between proNGF level and cognitive performance indices only for the intervention group for (1) planning sum score (*r* = −.95, *P* = .005); (2) working memory updating average index (*r* = −.96, *P* = .004); and (3) flexibility and originality dimensions of creative thinking (*r* = −.96, *P* = .004; *r* = −.95, *P* = .005, resp.). See Figures 5(a)–5(d). No significant correlation was found for fluency (*P* = 0.017), inhibition (*P* = .28), and attention (*P* = .13) for QMT. In addition, no significant correlations between NGF level and cognitive performance indices emerged at post-test for the control group (.37 ≥ *P* ≥ .17).

## 4. Discussion

### 4.1. Cognitive Effect of QMT

In exercise and cognition research in the last decades, scientists have progressively focused on the effects of physical activity on higher-level cognition, with major interest in how the quantitative parameters of exercise and their physical fitness outcomes moderate or mediate physical exercise effects on cognitive functioning [44]. Recently, it has been proposed to shift the focus toward the qualitative exercise characteristics to reap largest cognitive benefits from gross-motor cognitive training tasks that are both physically effortful and cognitively engaging [46]. This new approach bridges the areas of whole-body mental training [10, 12, 47] and exercise and cognition research [46, 48], with their intersection point being represented by a shared neuroscience perspective. The main aim of the current report was to study whole-body MBP-induced creativity enhancement, neurotrophic change, and the relation between them. To this end, we examined the effects of 4 weeks of daily Quadrato motor training (QMT) on creativity compared to a walking training in healthy adults (Study A). We found that, similar to the effects of a session of QMT [10], 4 weeks of daily QMT significantly increased ideational flexibility in healthy adults. A similar trend of improved creativity was observed in children, associated with the specific improvement of planning ability that shares with creativity metacognitive characteristics and of working memory, a core executive function involved in both creative thinking and planning (Study B).

### 4.2. Neurotrophic Effect of QMT

Neurotrophins, such as NGF, are central players in the functions of nervous system. They are involved in neuronal shaping and plasticity, as well as synaptic connectivity in the sympathetic and central nervous systems. Among neurotrophins, BDNF has been widely analyzed in correlation with physical training of various natures, mainly acute and moderate aerobic exercises. Several reports have highlighted variations in BDNF levels, following training, both in animal models [26, 27] and in humans [23, 24, 49]. In addition, it was reported that environmental enrichment aimed at stimulating cognition and learning, along with exercise, is a mean of increasing BDNF levels [27, 50]. These and many other reports point to a role for BDNF in mediating the beneficial effects of physical and mental practices on the individual's brain health and general well-being. To the best of our knowledge, analogous studies on NGF/proNGF in healthy humans have not been conducted. With the aim of filling this gap and examining the effects of MBP on proNGF, we have measured levels of salivary proNGF before and after 4 weeks of daily QMT in adults and children. The choice of analyzing proNGF is due to the fact that the mature form NGF is almost never detectable outside the cells, with the precursor being the prevalent form present in the extracellular space [21]. In both age groups we observed a trend of decreased proNGF following QMT. This trend may have significant theoretical and practical implications, as evidence of increased proNGF levels in Alzheimer's patients and in individuals with several degrees of cognitive impairment highlights the association of this factor with brain health [14, 22, 51]. A more recent research reported both increased proNGF and decreased BDNF levels in postmortem brain samples of subjects with several tauopathies (non-Alzheimer's disease dementias) as compared to age matched controls [52]. Interestingly, we may have also suggested an inverse relationship between proNGF and BDNF. More specifically, in a parallel longitudinal study with healthy participants, we have shown increased amounts of proBDNF following 3 months of daily QMT practice [53]. These results appear symmetrical to the ones conducted on the dementia patients, supporting previous claims for the inverse directionality of change in BDNF and proNGF following training [54]. As widely reported in the literature, NGF plays a pivotal role as a factor that helps regulate individual's responses to stress ([19], see [55] for a review). Taken together these concepts lead us to speculate that a first phase of stress reduction (i.e., decrease of proNGF and increased emotional well-being) could be prodromal to the subsequent increase of BDNF which, in turn, could correlate with increased neuroplasticity, considering the structural changes shown by MRI of brains of sitting or movement meditation [53, 56, 57].

From the molecular point of view the decrease may be explained in several ways: (i) less proNGF mRNA and/or protein is produced, (ii) less protein is secreted from the cells, (iii) processing and/or degradation of the precursor is enforced outside the cells, and (iv) binding of the molecule to its receptor and therefore its internalization are enhanced. At this stage it is not possible to distinguish among all this options and further work is needed to elucidate this point (i.e., by utilizing salivary cells to look for proNGF mRNA and/or protein levels).

### 4.3. Correlation between Change in ProNGF and Creativity

In adults, a negative correlation was found between change in flexibility and proNGF (Figure 3). The results of the corresponding analysis performed on children's data are consistent with the findings of the adult study: a negative correlation between proNGF and behavioral indices of cognitive functioning, related to the whole-body cognitive training intervention, was in fact observed (Figure 5). Together, these are novel results, since NGF/proNGF concentration has been largely neglected in exercise and cognition research aimed at identifying neurobiological markers of acute [58] and chronic [59] exercise effects on brain health and cognitive efficiency.

Nevertheless, in contrast to adults, in the case of children there was a significant negative correlation not between pre-post Δ values of proNGF and cognitive performances, but only between post-test values in the group that underwent the QMT. This finding may suggest that at developmental age, children's cognitive performance did not consistently vary as a function of exercise-induced changes in neurotrophic factors. Several factors may have influenced children's baseline cognitive performance and its changes over time, impeding to see a strict relationship between intervention-related changes in cognitive efficiency and their potential neurobiological marker. However, whatever the size of the neurobiological and functional effect of QMT, the whole-body cognitive training seems to “align” several types of cognitive performance to the concentration of a specific neurobiological marker. After this mindfulness training, there seems to be a prioritization of the neurobiological factor which seems to assume a major role as correlate of cognitive and metacognitive efficiency.

The extension of the present study to children, even though preliminary in nature with a small sample size, adds to the adult study because of the broader set of cognitive performances tested and the selectivity of the intervention-related association between cognitive performances and neurobiological factors. The correlational results suggest that a reduced proNGF level is a biological marker of the potential of whole-body cognitive training to enhance specific components of higher-level cognition. While in the last decades, the attention of exercise and cognition researchers [44] and neurodevelopmentalists [60] has focused on interventions aiding executive function, recently it has been proposed to go beyond this view and consider quality physical activity as enrichment aiding the development of metacognition [48]. Metacognition reflects the acquisition and use of declarative, procedural, and strategy knowledge to successfully deal with problem solving tasks. Tomporowski and colleagues [48] propose that quality physical activity interventions that are cognitively challenging would alter children's metacognitive processes through changes in executive function efficiency.

Interestingly, the present results are consistent with this novel view: significant correlations with the neurobiological marker after the QMT were found for a foundational executive function as well as for metacognitive functions such as planning and creativity that rely on, but neither overlap with, nor critically depend on, single executive functions [61, 62]. Instead, no significant correlation emerged between proNGF and executive attention performance or inhibition. These diverging results are not surprising, since inhibition seems not to be linked to planning in young children [63]. Moreover, the absence of association between executive attention and proNGF level is in line with evidence that some types of mindfulness practices enhance other subcomponents of the attention networks [64]. Therefore, the QMT seems to be a specific type of mindfulness physical activity that has the potential to influence the development of metacognition and to enhance executive functions [10, 12].

An alternative interpretation of the presence of significantly high (negative) correlations between proNGF concentration and cognitive performances may be a learning effect due to the short time between cognitive measurements before and after the intervention. Interindividual differences in learning slopes of cognitive tasks during the preliminary training trials may have dampened, at pre-test, the potential interrelation between cognitive efficiency and its neurobiological marker that may exist prior to any change induced by the whole-body cognitive training intervention. However, this interpretation is less probable, since the significant alignment of neurobiological and cognitive-behavioral data at post-test occurred for the intervention group only. Furthermore, tests as the RNG, showing no significant correlation with proNGF concentration at pre-test and a very high correlation at post-test, are reported to be stable against learning effects [65]. To exclude any influence of differential learning effects of cognitive test performance on the relationship of interest, future studies are warranted with participants acting as their own controls with two successive measurement time points before the intervention and one after it. In addition, due to the low power of the correlation analysis (*n* = 19 in Study A, *n* = 5 in Study B) and the nonsignificant effect of training on proNGF level, probably requiring longer duration, this finding should be treated as suggestive and should be tested in the future, using larger groups and longer training periods.

### 4.4. Limitations

The current study is a preliminary attempt to examine empirically the question of the connection between MBP-induced effects on creativity and neurotrophic change. Its main limitations are the small and unbalanced sample size. In the future, a study on a larger sample that includes additional training regimes with a similar level of engagement may extend the current results. Future studies should expand the age group under examination and combine the examination of electrophysiological and physiological effects of QMT.

### 4.5. Conclusions, Implications, and Future Directions

In conclusion, the current research emphasizes the importance of examining the neurobiological correlates of mindful-physical activity practices. The present results support the usefulness of integrating physical and mental training across the lifespan [46, 66] and suggest that exposure to holistic forms of environmental enrichment as the QMT aids the development of executive function [45] and metacognition [48]. The results also further our understanding of the underlying biological mechanisms, confirming and extending the concept that brain growth factors are central to the benefits of exercise [59]. In comparison to other MBP, the QMT has the advantage of being a training of relatively short duration (possibly several minutes) and that can be practiced in limited spaces. These unique aspects render the QMT a technique warranted for scientific exploration, with the future aim of implementing this technique in various health promoting and educational setups.

## References

[1] Chávez-Eakle R. A., Graff-Guerrero A., García-Reyna J.-C., Vaugier V., Cruz-Fuentes C. (2007). Cerebral blood flow associated with creative performance: a comparative study. *NeuroImage*.

[2] Sternberg R. J., Lubart T. I. (1996). Investing in creativity. *American Psychologist*.

[3] Benson H., Proctor W. (2003). *The Breakout Principle: How to Activate the Natural Trigger That Maximizes Creativity, Athletic Performance, Productivity and Personal Well-Being*.

[4] Boynton T. (2001). Applied research using alpha/theta training for enhancing creativity and well-being. *Journal of Neurotherapy*.

[5] Maslow A. H. (1963). The creative attitude. *The Structurist*.

[6] Reynolds F. (2005). The effects of creativity on physical and psychological well-being: current and new directions for research. *Teoksessa Schmid, Therese (toim.): Promoting Health through Creativity: For Professionals in Health, Arts and Education*.

[7] Colzato L. S., Ozturk A., Hommel B. (2012). Meditate to create: the impact of focused-attention and open-monitoring training on convergent and divergent thinking. *Frontiers in Psychology*.

[8] Domino G. (1977). Transcendental meditation and creativity: an empirical investigation. *Journal of Applied Psychology*.

[9] Otis L. S. (1974). The facts on transcendental meditation: III. If well integrated but anxious, try TM. *Psychology Today*.

[10] Ben-Soussan T. D., Glicksohn J., Goldstein A., Berkovich-Ohana A., Donchin O. (2013). Into the square and out of the box: the effects of quadrato motor training on creativity and alpha coherence. *PLoS ONE*.

[11] Ben-Soussan T. D., Glicksohn J., Berkovich-Ohana A. Inner view: attentional effort, mindfulness and altered states of consciousness experiences following Quadrato Motor Training.

[12] Ben-Soussan T. D., Berkovich-Ohana A., Glicksohn J., Goldstein A. (2014). A suspended act: increased reflectivity and gender-dependent electrophysiological change following Quadrato Motor Training. *Frontiers in Psychology*.

[13] Moretti D. V., Babiloni C., Binetti G. (2004). Individual analysis of EEG frequency and band power in mild Alzheimer's disease. *Clinical Neurophysiology*.

[14] Peng S., Wuu J., Mufson E. J., Fahnestock M. (2004). Increased proNGF levels in subjects with mild cognitive impairment and mild Alzheimer disease. *Journal of Neuropathology and Experimental Neurology*.

[15] Hamburger V., Levi-Montalcini R. (1949). Proliferation, differentiation and degeneration in the spinal ganglia of the chick embryo under normal and experimental conditions. *The Journal of experimental zoology*.

[16] Bibel M., Barde Y.-A. (2000). Neurotrophins: key regulators of cell fate and cell shape in the vertebrate nervous system. *Genes and Development*.

[17] Aloe L., Alleva E., Bohm A., Levi-Montalcini R. (1986). Aggressive behavior induces release of nerve growth factor from mouse salivary gland into the bloodstream. *Proceedings of the National Academy of Sciences of the United States of America*.

[18] Levi-Montalcini R., Aloe L., Alleva E. (1990). A role for nerve growth factor in nervous, endocrine and immune system. *Progress in Neuroendocrinoimmunology*.

[19] Castrén E., Võikar V., Rantamäki T. (2007). Role of neurotrophic factors in depression. *Current Opinion in Pharmacology*.

[20] Chao M. V. (2003). Neurotrophins and their receptors: a convergence point for many signalling pathways. *Nature Reviews Neuroscience*.

[21] Lu B., Pang P. T., Woo N. H. (2005). The Yin and Yang of neurotrophin action. *Nature Reviews Neuroscience*.

[22] Fahnestock M., Michalski B., Xu B., Coughlin M. D. (2001). The precursor pro-nerve growth factor is the predominant form of nerve growth factor in brain and is increased in Alzheimer's disease. *Molecular and Cellular Neuroscience*.

[23] Zoladz J. A., Pilc A. (2010). The effects of physical activity on the brain-derived neurotrophic factor: from animal to human studies. *Journal of Physiology and Pharmacology*.

[24] Rasmussen P., Brassard P., Adser H. (2009). Evidence for a release of brain-derived neurotrophic factor from the brain during exercise. *Experimental Physiology*.

[25] Cai J., Hua F., Yuan L. (2014). Potential therapeutic effects of neurotrophins for acute and chronic neurological diseases. *BioMed Research International*.

[26] Langdon K. D., Corbett D. (2012). Improved working memory following novel combinations of physical and cognitive activity. *Neurorehabilitation and Neural Repair*.

[27] Bechara R. G., Kelly Á. M. (2013). Exercise improves object recognition memory and induces BDNF expression and cell proliferation in cognitively enriched rats. *Behavioural Brain Research*.

[28] Criscimagna-Hemminger S. E., Donchin O., Gazzaniga M. S., Shadmehr R. (2003). Learned dynamics of reaching movements generalize from dominant to nondominant arm. *Journal of Neurophysiology*.

[29] Donchin O., Francis J. T., Shadmehr R. (2003). Quantifying generalization from trial-by-trial behavior of adaptive systems that learn with basis functions: theory and experiments in human motor control. *The Journal of Neuroscience*.

[30] Guilford J. P. (1950). Creativity. *The American Psychologist*.

[31] Guilford J. P., Christensen P. R., Merrifield P. R., Wilson R. C. (1978). *Alternate Uses: Manual of Instructions and Interpretation*.

[32] Fink A., Graif B., Neubauer A. C. (2009). Brain correlates underlying creative thinking: EEG alpha activity in professional vs. novice dancers. *NeuroImage*.

[33] Netz Y., Tomer R., Axelrad S., Argov E., Inbar O. (2007). The effect of a single aerobic training session on cognitive flexibility in late middle-aged adults. *International Journal of Sports Medicine*.

[34] Runco M. A. (1986). Flexibility and originality in children's divergent thinking. *The Journal of Psychology*.

[35] Guilford J. P. (1968). *Creativity, Intelligence, and Their Educational Implications*.

[36] Russ S. W. (1998). Play, creativity, and adaptive functioning: implications for play interventions. *Journal of Clinical Child Psychology*.

[37] Bruno M. A., Cuello A. C. (2006). Activity-dependent release of precursor nerve growth factor, conversion to mature nerve growth factor, and its degradation by a protease cascade. *Proceedings of the National Academy of Sciences of the United States of America*.

[38] Chang Y. K., Labban J. D., Gapin J. I., Etnier J. L. (2012). The effects of acute exercise on cognitive performance: a meta-analysis. *Brain Research*.

[39] Torrance E. P. (1989). *Test di Pensiero Creativo*.

[40] Towse J. N., McLachlan A. (1999). An exploration of random generation among children. *British Journal of Developmental Psychology*.

[41] Towse J. N., Neil D. (1998). Analyzing human random generation behavior: a review of methods used and a computer program for describing performance. *Behavior Research Methods, Instruments, and Computers*.

[42] Naglieri J. A., Das J. P. (1997). *Cognitive Assessment System*.

[43] Das J. P., Naglieri J. A., Kirby J. R. (1994). *Assessment of Cognitive Processes: The PASS Theory of Intelligence*.

[44] Etnier J. L., Chang Y.-K. (2009). The effect of physical activity on executive function: a brief commentary on definitions, measurement issues, and the current state of the literature. *Journal of Sport & Exercise Psychology*.

[45] Diamond A., Lee K. (2011). Interventions shown to aid executive function development in children 4 to 12 years old. *Science*.

[46] Pesce C. (2012). Shifting the focus from quantitative to qualitative exercise characteristics in exercise and cognition research. *Journal of Sport and Exercise Psychology*.

[47] Ben-Soussan T. D., Avirame K., Glicksohn J., Goldstein A., Harpaz Y., Ben-Shachar M. (2014). Changes in cerebellar activity and inter-hemispheric coherence accompany improved reading performance following Quadrato Motor Training. *Frontiers in Systems Neuroscience*.

[48] Tomporowski P. D., McCullick B., Pendleton D. M., Pesce C. Exercise and children's cognition: the role of task factors and a place for metacognition.

[49] Brunelli A., Dimauro I., Sgrò P. (2012). Acute exercise modulates BDNF and pro-BDNF protein content in immune cells. *Medicine and Science in Sports and Exercise*.

[50] Baroncelli L., Braschi C., Spolidoro M., Begenisic T., Sale A., Maffei L. (2010). Nurturing brain plasticity: impact of environmental enrichment. *Cell Death and Differentiation*.

[51] Wang X. X., Tan M. S., Yu J. T., Tan L. (2014). Matrix Metalloproteinases and their multiple roles in Alzheimer’s disease. *BioMed Research International*.

[52] Belrose J. C., Masoudi R., Michalski B., Fahnestock M. (2014). Increased pro-nerve growth factor and decreased brain-derived neurotrophic factor in non-Alzheimer's disease tauopathies. *Neurobiology of Aging*.

[53] Ben-Soussan T. D., Piervincenzi C., Venditti S., Verdone L., Caserta M., Carducci F. (2015). Increased cerebellar volume and BDNF level following sensorimotor training. *Synapse*.

[54] Angelucci F., Ricci E., Padua L., Sabino A., Tonali P. A. (2007). Music exposure differentially alters the levels of brain-derived neurotrophic factor and nerve growth factor in the mouse hypothalamus. *Neuroscience Letters*.

[55] Aloe L., Alleva E., Fiore M. (2002). Stress and nerve growth factor: findings in animal models and humans. *Pharmacology Biochemistry and Behavior*.

[56] Hölzel B. K., Carmody J., Vangel M. (2011). Mindfulness practice leads to increases in regional brain gray matter density. *Psychiatry Research: Neuroimaging*.

[57] Wei G.-X., Xu T., Fan F.-M. (2013). Can Taichi reshape the brain? A brain morphometry study. *PLoS ONE*.

[58] Ferris L. T., Williams J. S., Shen C.-L. (2007). The effect of acute exercise on serum brain-derived neurotrophic factor levels and cognitive function. *Medicine and Science in Sports and Exercise*.

[59] Cotman C. W., Berchtold N. C., Christie L.-A. (2007). Exercise builds brain health: key roles of growth factor cascades and inflammation. *Trends in Neurosciences*.

[60] Diamond A. (2013). Executive functions. *Annual Review of Psychology*.

[61] Dietrich A., Kanso R. (2010). A review of EEG, ERP, and neuroimaging studies of creativity and insight. *Psychological Bulletin*.

[62] Pennequin V., Sorel O., Fontaine R. (2010). Motor planning between 4 and 7 years of age: changes linked to executive functions. *Brain and Cognition*.

[63] Carlson S. M., Moses L. J., Claxton L. J. (2004). Individual differences in executive functioning and theory of mind: an investigation of inhibitory control and planning ability. *Journal of Experimental Child Psychology*.

[64] Jha A. P., Krompinger J., Baime M. J. (2007). Mindfulness training modifies subsystems of attention. *Cognitive, Affective & Behavioral Neuroscience*.

[65] Towse J. N., Valentine J. D. (1997). Random generation of numbers: a search for underlying processes. *European Journal of Cognitive Psychology*.

[66] Curlik D. M., Shors T. J. (2013). Training your brain: do mental and physical (MAP) training enhance cognition through the process of neurogenesis in the hippocampus?. *Neuropharmacology*.

